# Intercultural Friendships with International Students in China: Examining the Role of Intergroup Contact, Intercultural Communication Competence, Host Country Nationals’ Attitudes, and Perceived Intergroup Threats

**DOI:** 10.3390/bs13100855

**Published:** 2023-10-18

**Authors:** Lingjie Tang, Chang’an Zhang

**Affiliations:** School of Foreign Studies, Xi’an Jiaotong University, Xi’an 710049, China; zca65@163.com

**Keywords:** host country nationals, international students, intercultural friendships, intergroup contact, intercultural communication competence, intergroup attitudes, perceived intergroup threats

## Abstract

International students studying and living in a foreign context often complain about difficulties establishing friendships with host nationals. This study investigates host country nationals’ (HCNs) willingness to develop intercultural friendships with international students who are sojourning in China by exploring the effects of face-to-face and online intergroup contact, HCNs’ attitudes, intercultural communication competence (ICC), and perceived intergroup threats. Survey data from 469 HCNs indicate that (a) face-to-face and online contact are indirectly and positively related to their willingness to form intercultural friendships, (b) face-to-face contact can moderate the relationships of online contact with HCNs’ intergroup attitudes and perceived intergroup threats, and (c) both ICC and intergroup attitudes can positively predict friendship formation whereas perceived intergroup threats act as a negative predictor. The implications of our findings for future research and practice are presented.

## 1. Introduction

In recent years, China’s education system has undergone a major transformation as the internationalization of higher education has taken hold, which has led to China evolving into one of the world’s largest and most promising higher education systems across the globe [1]. According to the statistical report released by the Chinese Ministry of Education (MOE), a total of 492,185 foreign students from 196 countries/areas pursued their studies in China’s colleges and universities in 2018 prior to the outbreak of COVID-19 in 2019, marking an increase of 51.6% from 2009 [2]. The overseas student population on Chinese college campuses is diverse in many ways, as they come from different countries and races with distinct cultural norms, customs, and linguistic backgrounds. Foreign students are often regarded as notably diverse groups with respect to their physical features and racial identity (e.g., skin color, wearing styles, facial characteristics, etc.), their cultural habits and norms of conduct, their religious beliefs and political belonging (e.g., Buddhism, Christian, Democrat, etc.), and the languages they speak [3]. Individuals may feel nervous and awkward when socializing with those who are culturally different [4], in part due to low levels of ICC. In addition, when interacting with non-native speakers of a language, some may undergo adverse emotions, such as impatience and agitation. Others might lack the motivation and desire to befriend culturally different individuals [5]. 

Interacting and establishing friendships with foreign students can, however, have tremendous benefits not only for the culturally different but also for host nationals. As for foreign students, intercultural friendships with host nationals are beneficial not only in reducing prejudice [6], but also in resulting in greater language skills, better academic performance, higher levels of life satisfaction, reduced psychological stress, decreased homesickness, and a better image of the host country [7]. Similarly, students of the host country can acquire valuable intercultural communication skills, gain a broader global perspective, and form a diverse friendship network. In light of increasing cultural diversity and demographic shifts worldwide, there is a growing focus on intergroup relations and intercultural attitudes [8,9]. However, few articles from the literature can be found on the subject of intercultural friendships [10,11]. A large proportion of the existing literature regarding intercultural friendships focused on acculturating outgroup members (e.g., immigrants and international students), neglecting the important roles of host nationals. In particular, it seems unclear how Chinese students interact and form relationships with foreign students. It is, therefore, crucial to explore the extent to which Chinese students, as host nationals, interact with these international students from various continents. 

Although the context of adjustment is clearly important, there is still variability in whether individuals will befriend international students. Researchers have started to shift their attention towards the underlying motivations and interests of host nationals. Studies on intercultural relations suggest that a number of factors may influence intercultural friendship formation, including ICC, perceived intergroup threats, and intercultural attitudes, which may be beneficial to consider [9,12]. ICC and perceived intergroup threats as antecedents of intercultural attitudes should be especially relevant in multicultural settings, the context of higher education in China, where intercultural interaction is virtually unavoidable due to the enrollment of an increasing number of international students [13]. Although the two variables have been found to play important roles in affecting intercultural attitudes [14,15], little is known about how these variables interact with intergroup contact and predict host nationals’ willingness to form friendships with the culturally different. Furthermore, to gain more insights into the topic, we distinguish between offline contact, which is direct face-to-face contact, and online contact, which is indirect contact via social networking sites. Differentiating the roles of the two forms of contact is important as they have distinctive communication styles and contextual features. Meanwhile, recent research suggests that it is important to closely examine the mechanisms for joint work between direct and indirect contact [16,17]. Therefore, our study continues this research line by testing how both forms of intergroup contact predict willingness to construct intercultural friendships. 

Thus, the focus of this study has been on four areas: intergroup contact, ICC, perceived intergroup threats, and HCNs’ intergroup attitudes. Our aim with this study was to explore host national perspectives, their intercultural attitudes and contact experiences with international students in particular. To the best of our knowledge, previous research has seldom examined intercultural friendships between HCNs and international students in the context of China, and only a small number of studies have touched upon the research topic across the world [18,19]. Therefore, it is of immediate and vital significance to explore these factors possibly affecting HCNs’ willingness to interact and foster intercultural bonds with international students in China. 

### 1.1. Intercultural Friendship Formation

Friendship represents a distinctive and significant form of interpersonal connection. Its uniqueness lies in its voluntary nature and the emphasis on viewing individuals as complete beings rather than merely occupants of roles [20]. Intercultural friendships are characterized by differences between cultural groups that offer both rewards and challenges. These friendships involve mutual trust, understanding, and social interactions that extend beyond mere contact. To comprehend the dynamics of forming intercultural friendships, Kudo and Simkin [21] introduce two social–psychological perspectives. First, they highlight how friendships differ across cultures in aspects such as scope, commitment, duration, and mutual trust. Second, they emphasize that intracultural and intercultural friendships offer distinct experiences. Intercultural friendships epitomize a profound convergence between the personal and cultural dimensions of communication processes [18]. These processes require individuals to navigate variations in cultural values and languages while dispelling enduring stereotypes. Yet, in the process, they acquire exclusive cultural insights, broaden their perspectives, and shatter stereotypes and prejudices. However, diverse cultural groups operate under distinct systems of meaning, which complicates the sharing of information, thus elevating the likelihood of misunderstandings and threats, uncertainty, frustration, and conflicts [22]. These obstacles pose significant challenges to both the establishment and sustenance of intercultural friendships. Nevertheless, it is important to note that despite these formidable challenges, individuals continue to forge and uphold enriching and long-lasting intercultural friendships. 

It is important to acknowledge that the term “intercultural” can encompass individuals with various demographic characteristics, such as gender, age, and physical ability as well as nationality and ethnicity [23]. However, for the scope of this study, we adopt a specific definition of “intercultural”, with a focus on cultural differences and on individuals of different nationalities—international students. 

Researchers have identified a multitude of factors that play significant roles in shaping the development of intercultural friendships. These factors include cultural differences, individual personalities, intercultural competence, the frequency of intergroup contact [10], different notions of friendship, and intergroup attitudes [24,25]. Meanwhile, previous research has revealed that numerous challenges and barriers impede the development of intercultural friendships, and while there are similarities in how individuals perceive friendships, significant distinctions exist that hinder intercultural friendship formation. Perceived threats, prejudice, and discrimination [26] as well as negative intercultural attitudes from host nationals [27] are viewed as common challenges for intercultural friendship development. Moreover, language barriers and cultural distance have also been found to affect intercultural friendship formation [28,29]. 

### 1.2. Major Factors Affecting Intercultural Friendship Formation

#### 1.2.1. Intergroup Contact 

Originating from the period of racial segregation in the U.S., the contact hypothesis has a simple and intuitive core: Frequent and positive encounters with individuals from a different group can enhance one’s overall judgments of that group and diminish the prejudiced attitudes towards it, which would be beneficial for the formation of intercultural friendships. Extensive studies have revealed that despite existing disparities in group status, intergroup contact has the potential to mitigate unfavorable attitudes harbored by members of the predominant group towards marginalized groups, such as ethnic minorities, international sojourners, homosexuals, and Muslims [30,31]. 

In addition, as evidenced by intergroup contact theory [32], intergroup contact with outgroup members may help decrease prejudice and conflict and may foster positive intergroup attitudes. This can be explained by the fact that intergroup contact helps foster favorable feelings for, knowledge about, and empathy toward the conditions and problems of the outgroup members and potentially diminishes intergroup apprehension and fosters affirmative intercultural dispositions and more positive attitudes [33], which, in turn, engenders a greater appreciation for diversity and multiculturalism. Decades of research on intergroup contact have also found that perceived intergroup threats can be reduced through frequent interactions, thereby exerting positive indirect effects on intergroup attitudes and mitigating harmful, discriminatory behaviors [34]. Intergroup contact, whether it is online or offline, presents individuals with opportunities for self-disclosure and access to intercultural friendship circles [35]. In other words, intergroup contact can help create fertile ground for friendship formation. 

The role of face-to-face intergroup contact has been a central theme in the literature regarding intergroup relations and remains a significant area of focus. As evidenced by the anxiety/uncertainty management theory [36], the contact hypothesis, and its extensions, face-to-face contact functions as an effective strategy for lowering levels of intergroup threats and prejudice and reinforcing intergroup relations among different social groups [37]. Face-to-face contact was revealed to be effective in increasing intercultural knowledge [38], which was identified as a major factor for intergroup uncertainty management, thus improving individuals’ intergroup attitudes and feelings and increasing their interest in forming friendships. 

Compared to face-to-face contact in physical contexts, intergroup contact taking place in virtual online contexts can have a series of advantages. The contrast between face-to-face and online intergroup contact is noteworthy as online contact not only enhances existing relationships, but also provides an avenue for individuals to connect with a diverse range of new people who may be beyond their physical reach [39]. People may show a stronger tendency to engage in greater self-disclosure and more intimate interactions through online contact with outgroup members [40]. Drawing upon the social information processing theory [41], some scholars contend that prolonged online contact can foster a strong sense of interpersonal attraction and cultivate meaningful relationships as users creatively craft their communicative messages online, which can lead to positive effects on intergroup attitudes [42]. Social media platforms (e.g., WeChat, widely used in China, or Facebook and Instagram, widely used elsewhere) can provide their users with more openness and a greater sense of control during online intergroup encounters, which may reduce their possible perceptions of threats and the emergence of stereotypic attitudes. It is important to note that the use of social media platforms, such as WeChat, is becoming prevalent among the international student community, as evidenced by previous studies [43,44,45]. Therefore, online contact between Chinese and international students can take place via these popular social media platforms, providing an important channel for intergroup interactions.

#### 1.2.2. Intercultural Communication Competence 

A second important factor influencing intercultural friendship establishment is ICC, specifically those elements that bridge the gap between the initial phase of relationship building (where cultural complexities are obviously apparent) and the subsequent phase of stable interpersonal engagement (where cultural differences generally fade into the background) [46]. Scholars have rightly argued that ICC, as an umbrella term, is composed of three dimensions: cognitive, affective, and behavioral [47]. Previous research has found that many cognitive, affective, and behavioral intercultural competencies, such as cultural self-awareness, culture-specific knowledge, motivation, empathy, open-mindedness, language skills, and appreciation of difference [48], are applicable to intercultural friendship contexts. 

A series of studies have shown that ICC plays a superlative role in initiating intercultural friendships [19,49] and that competency in the various skills of ICC is recognized as one of the key predictors of intergroup attraction and smooth interaction [19]. For example, ICC is indispensable for the gathering of invaluable insights into diverse cultures and, therewith, promoting the acquisition of intercultural knowledge [50], which involves a nuanced comprehension of the fundamental components of deep culture as well as the ability to seamlessly integrate them into one’s daily life [19]. Host nationals’ deep understanding of different cultures is critical for intergroup interactions and relationship building. Meanwhile, extensive research has revealed that possessing exceptional communicative adaptability, which is an important aspect of ICC and refers to a blend of effective communication abilities and commendable personal traits [51], can remarkably enhance the contentment of individuals regarding the quantity and quality of their intercultural friendships [27]. 

Furthermore, the likelihood of intercultural friendship formation is also affected by some other aspects of ICC, such as motivation, empathy, and communication skills. Motivation is important for developing friendships, but some obstacles may diminish their impact. Studies have revealed that the preexisting conational networks into which one is born may lessen the necessity and motivation for individuals to establish intercultural bonds with others [52]. The communication strategies commonly used in East Asia, such as implicit communication in China, are effective within existing social networks but not useful for starting friendships between people from different cultures. Moreover, East Asian students in their repertoire often lack the communication skills (e.g., small talk) that are essential for building friendships with people from Western countries [53], necessitating consistent exposure and practice to bridge this gap. As one of the independent variables concerning ICC, empathy facilitates mutual understanding and tolerance between HCNs and outgroup members. It may be of significant relevance to intercultural friendships between Chinese host nationals and international students, many of whom have quite distinctive cultures, norms, and values and may compete for scarce resources (e.g., scholarships and employment). 

#### 1.2.3. Perceived Intergroup Threats and HCNs’ Attitudes

According to intergroup threat theory, both realistic and symbolic perceived threats negatively and concomitantly (or not) influence attitudes toward culturally different others, which may, in turn, affect individuals’ interest in intercultural friendship formation. Realistic threats stem from the scarcity of resources, such as employment opportunities, access to health care and state support, and food supply, which is why they are often referred to as economic threats [54,55]. Realistic threats can arise when there is a conflict between groups, competition for limited resources, or when the safety of those within the group is at risk [56]. Symbolic threats pose a danger to the fundamental principles of the host community, including their customs, traditions, convictions, and ethics. Such threats can undermine the very foundation of their way of life and the national culture and identity [54,55]. 

Through the lens of social psychology, foreign students can be viewed as outgroup members, distinct from the local in-group, and the recognition of international students as a minority group encompasses a complex interplay of respect and resentment, involving both favorable and unfavorable attitudes and sentiments. They could potentially be perceived as unwelcome rivals who are depriving local students of invaluable academic and material assets (such as access to prestigious academic programs, lodging amenities, financial assistance, job prospects, etc.). Consequently, it is possible for Chinese nationals to object to institutional policies and initiatives created to aid international students, and perceptions of realistic threats could potentially fuel intergroup hostility, lowering the possibilities of intercultural friendship formation. Accordingly, we reasoned that Chinese students may regard foreign students as a possible threat to their power, status, and access to valuable resources and opportunities within their educational institutions. 

While international students undoubtedly bring cultural and intellectual richness to the university community, their values, norms, and behavioral patterns may differ greatly from those of domestic students, leading to potential conflicts [3]. Therefore, we proposed that foreign students in China are possibly regarded as posing a symbolic threat to normative beliefs, values, and worldviews held by many Chinese students due to the perceptible cultural contrasts that many of these students exhibit on these dimensions. In addition, international students may have opinions that conflict with the beliefs of domestic students, and they may embody cultural, religious, and political ideologies that are not well-regarded within the host country [57]. 

Several studies have noted that both types of perceived threats can result in prejudiced attitudes and bias towards social outgroups, although the extent of their impact may differ depending on the particular group under consideration [58]. For instance, a study addressing the perceptions and attitudes of Americans and Mexicans towards each other has revealed that the perceived threats were key indicators of their attitudes in both groups [59]. Similarly, in a study by Schweitzer et al. [60], the researchers found that Australian college students who perceived both types of threats from different groups and refugees were more likely to hold negative anti-refugee attitudes. Therefore, perceived threat reduction and improved attitudes can be the prerequisite for intercultural friendship formation.

### 1.3. The Current Study and Hypothesized Model

**Figure 1:**
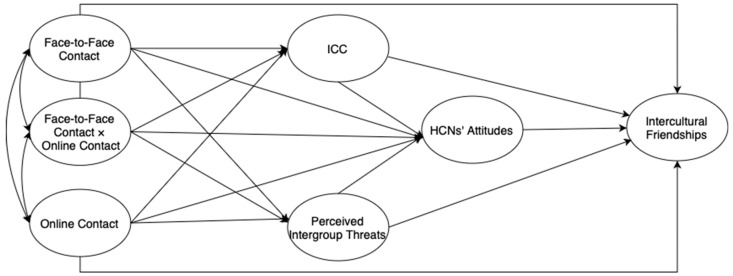
The conceptual research model.

Drawing upon previous studies [9,12,21] and theoretical underpinnings of the constructs closely inspected above, the present study draws on several factors to enhance our understanding of the interplay between HCNs and international students. Specifically, the study aims to explore (a) HCNs’ attitudes toward international students, (b) how inclined HCNs are to foster intercultural friendships with international students, (c) HCNs’ intergroup contact with international students, and (d) how HCNs’ ICC, attitudes toward international students, and perceived threats arising from their intergroup contact mediate the relationship between intergroup contact and intercultural friendships. Accordingly, the hypothesized model with relevant path directions is illustrated in Figure 1 in light of the following hypotheses we aim to test.

Concerning the moderating effect:

**H1.**  *Face-to-face contact will moderate the relationships of online contact with ICC (H1a), perceived intergroup threats (H1b), HCNs’ attitudes (H1c), and intercultural friendships (H1d). Specifically, low levels of face-to-face contact will predict stronger relationships between these variables, whereas high levels of face-to-face contact will predict weaker or non-existent relationships between these variables.*

Concerning the mediating effects:

**H2.**  *ICC, perceived intergroup threats, and HCNs’ attitudes will mediate the relationship between face-to-face contact and intercultural friendships.*

**H3.**  *ICC, perceived intergroup threats, and HCNs’ attitudes will mediate the relationship between online contact and intercultural friendships.*

## 2. Methods

### 2.1. Participants

Since this study targeted HCNs who previously had intergroup contact experiences with international students, a question was raised about whether an individual had such experience prior to the survey. Only those who responded “yes” were given the chance to fill out the questionnaire. A total of 469 Chinese students from 31 universities and colleges participated in the present study, including 206 (44%) males and 263 (56%) females. An amount of 364 students (78%) were affiliated with Project 985/211 universities, a group of prestigious institutions in China receiving significant government funding and support to enhance their academic excellence and research capabilities with the goal of becoming world-class universities [61,62]. Our interest in this indicator is because Project 985/211 universities in China are generally believed to accommodate a higher population of international students from a broader range of countries/regions compared to non-985/211 universities, thereby providing more intergroup contact opportunities for HCNs. It was essential to test this factor to assess its potential impact on the formation of intercultural friendships between HCNs and international students. The most common age range was 24–26 years (*n* = 177; 38%), followed by 21–23 years (*n* = 150; 32%), 18–20 years (*n* = 127; 27%), and 27 years or older (*n* = 15; 3%). The sample comprised 415 (88%) undergraduate students and 54 (12%) graduate students (including nine doctoral students). Among them, 106 (23%) responded that they had prior experience studying in another country. 

### 2.2. Procedures

Participants were recruited to take part in the study lasting from December 2022 to March 2023. This study was conducted in line with the stipulations of the World Medical Association Declaration of Helsinki Ethical Principles [63]. Once participants agreed to voluntarily take part in the research based on the principles of anonymity and confidentiality, they were given access to an online survey questionnaire, which they could complete at their convenience. All participants were presented with an informed consent form that outlined the purposes and procedures of the study. Upon consenting to the conditions of the study, the participants were able to complete the questionnaire, which took them approximately 15 to 20 min. The self-report survey questionnaire was originally written in English and translated into Chinese with back translation done by a professional bilingual translator and then reviewed by two professors and several Chinese doctoral students to ensure the equivalence of meaning. The selection of measurement scales was made with great consideration for their reliability and validity, ensuring their utmost quality and accuracy. 

### 2.3. Statistical Analyses

In the study, data analysis was performed using SPSS 26 and AMOS 26. As the first step, SPSS was used to conduct preliminary analyses, compute descriptive statistics, and estimate intercorrelations among various variables. Then, the structural model was used to test the research hypotheses. In terms of the structural model evaluation, “Maximum Likelihood” and some fit indices frequently chosen by scholars were taken into account. The model’s goodness of fit should have to meet the following criteria to reach acceptability: χ^2^/df < 3; CFI, IFI, and TLI ≥ 0.90; RMSEA and SRMR ≤ 0.08 [64]. Moreover, we examined mediation by evaluating and estimating the statistical significance of indirect effects. To this end, we employed the bootstrapping technique proposed by Shrout and Bolger [65] by means of generating 5000 bootstraps and calculating upper and lower limit confidence intervals. In addition, the simple slope analysis was employed to evaluate the moderating effects [66].

### 2.4. Measures

#### 2.4.1. Intergroup Contact

##### Face-to-Face Contact

As an important measure of intergroup contact, participants responded to three items adapted from Biernat and Crandall [67] in order to measure the frequency of their face-to-face contact with international students in China. These statements were rated on a seven-point response scale anchored by 1 (never) and 7 (always), with higher scores meaning closer intergroup contact with the international community. Sample statements on the scale were: (a) “How often do you talk to and engage in informal face-to-face conversations with foreign students?” and (b) “How often do you do things socially with international students? (This includes things like watching movies, attending parties, dining together, etc.)”. 

##### Online Contact

Four items adapted from previous studies [68] were utilized to measure the frequency and amount of online communication by respondents interacting with international students in China. Specifically, one statement asked about the number of days of online contact in the past week. The response categories were arranged as 0 (none), 1 (1–2 days), 2 (3–4 days), 3 (5–6 days), and 4 (every day). The remaining three items inquired about the approximate duration of online communication on the last day, on an average weekday, and on an average weekend, which was coded as 1 (less than 15 min), 2 (between 15 min and 1 h), 3 (1–2 h), 4 (3–4 h), and 5 (more than 4 h). Since these items were anchored with different values, responses to the items were averaged to produce the mean score, with higher scores corresponding to higher amounts of online intergroup contact with international students. This scale has been adopted and validated in previous studies, generating acceptable internal consistency [17,69]. 

##### ICC

As a measure of ICC, participants responded to a ten-item Likert scale with the continuum ranging from 1 (strongly disagree) to 7 (strongly agree) [70], and higher scores corresponded to greater ICC. The scale consisted of three items on cognitive aspects, four items on affective aspects, and three items on behavioral aspects. Sample items included (a) “I feel more comfortable with people from my own culture than with people from other cultures” and (b) “Most of my friends are from my own culture”. This measure had high test–retest reliability (Cronbach’s alpha = 0.77) and had also been utilized in a variety of intercultural studies [71]. 

#### 2.4.2. HCNs’ Intergroup Attitudes toward International Students 

To measure participants’ overall evaluation of foreign students, an attitude assessment scale adapted from Ward et al. [72] was completed. Participants indicated their attitudes toward international students using a 12-statement measure ranging from 1 (strongly disagree) to 7 (strongly agree), with higher scores reflecting a more favorable attitude toward the group. Example items are: (a) “I don’t like international students” and (b) “International students are good role models”. This instrument, as demonstrated by relevant studies in a variety of contexts [73], possesses high test–retest reliability and has been applied to measure intergroup attitudes toward a broad range of groups. 

#### 2.4.3. Perceived Intergroup Threats

The measurement of perceived intergroup threats encompassed nine items strategically designed to capture various types of threats that hold contextual significance within the realm of Chinese universities. This scale was adapted from Stephan and Stephan [56] in their research on examining the prediction of prejudice based on three separate studies. Participants’ perception of threats was rated on a seven-point Likert scale with endpoints 1 (strongly disagree) and 7 (strongly agree). Five adapted items were used to judge the perception that foreign students pose a potential symbolic threat to the norms, values, and culture of the majority Chinese student population. Meanwhile, participants responded to another four items to gauge their perceptions of realistic threats international students may pose to their academic and economic well-being and their social status. Exemplary statements include: (a) “International students take jobs away from Chinese students (e.g., on-campus employment, teaching/research assistantships)” and (b) “Cherished Chinese norms and traditions are threatened somewhat by increasing international student enrollment in Chinese campuses”. The nine items capturing different types of threats were highly correlated. Higher scores reflect greater intergroup threat perceptions. 

#### 2.4.4. Intercultural Friendships

The diversity of respondents’ friendship networks in terms of free time, study sessions, and recreation activities was used to measure intercultural friendships. Participants indicated the extent to which they socialized with international students using the six-item scale adapted from Glass et al. [74]. Their responses were gauged on a seven-point Likert-type scale from 1 (strongly disagree) to 7 (strongly agree). The original scale produced reliable internal consistency and was modified to be more appropriate for the present research. Sample items include: (a) “In study sessions, I tend to study with international students” and (b) “I tend to participate in recreation activities with international students”. 

## 3. Results

### 3.1. Validity, Construct Reliability, and Common Method Bias Control

We conducted Harman’s single factor test, suggested by Podsakoff et al. [75], due to self-reported data that may lead to common method bias. All 44 items of the six variables were assigned to a single un-rotated factor, but around 37% of the total variance was captured by this factor, which is below the recommended threshold of 40%. This suggests that a significant portion of the variance was not accounted for by a single factor. Then, a single-factor model was evaluated, yielding a rather poor model fit: χ^2^ /df = 7.366, *p* < 0.001; CFI = 0.578; IFI = 0.580; TLI = 0.558; SRMR = 0.107; RMSEA = 0.117 (90% confidence interval (CI) for RMSEA = [0.114, 0.119]). The combined findings unequivocally demonstrate that the current study was unaffected by any potential common method bias. 

Additionally, we performed a confirmatory factor analysis (CFA) to assess scale construct validity. We followed the Fornell–Larcker’s [76] criterion, with acceptable benchmarks for factor loadings (>0.60), composite reliability (CR) (>0.60), and average variance extracted (AVE) (>0.50). Overall, our analysis of the major study variables demonstrated relatively strong factor loadings (0.603–0.914), high CR values (0.899–0.943), and acceptable AVE values (0.510–0.791), confirming satisfactory convergent validity. Discriminant validity was assessed by comparing the square root ($\sqrt{AVE}$) with construct intercorrelations, following Fornell et al.’s [77] methodology. The results indicated that $\sqrt{AVE}$ (0.714–0.889) for each dimension exceeded the construct correlations, affirming robust discriminant validity.

### 3.2. Preliminary Analyses 

Means, standard deviations, Cronbach’s α coefficients, and the zero-order correlation matrix for all of these focal variables, along with relevant demographic variables in this sample, are presented in Table 1. Cronbach’s alpha coefficients for the measures range from a low of 0.842 for perceived intergroup threats to a high of 0.943 for HCNs’ attitudes, indicating that all measures in the study have internal consistency ranging from acceptable to strong.

Examining descriptive statistics indicated that HCNs’ attitudes toward foreign students in this sample are relatively positive and favorable as the mean score (M = 4.54) is above the midpoint of the scale, suggesting that 77% (midpoint ≥ 4) of Chinese college students in this sample held generally positive attitudes toward culturally different students. This mean evaluative score is comparable to that obtained for international students [12]. As for respondents’ ICC (M = 4.66), its mean score is above the midpoint of the scale, suggesting that 83% of respondents have a relatively satisfactory ability in the measure. The mean score for the intercultural friendship measure (M = 4.84) suggests that 84% of Chinese college students in the study were willing to interact and form relationships with their international peers. In terms of intergroup contact with culturally different others, participants reported slightly lower levels of online contact (M = 3.14 or 65%; midpoint ≥ 3) as compared with face-to-face contact (M = 4.24 or 67%) with international students. Participants scored below the midpoint on the scale of perceived intergroup threats (M = 3.25), indicating that international students were generally viewed as competitors by 30% of students in the sample, whereas 26% regarded them as posing potential threats to Chinese traditions, norms, and cultures.

Zero-order correlation coefficients aligned with our anticipated direction. As it is presented in Table 1, it was found that in both types of intergroup contact, face-to-face contact was positively related to HCNs’ attitudes (r = 0.406, *p* < 0.01), ICC (r = 0.391, *p* < 0.01), and intercultural friendships (r = 0.426, *p* < 0.01) but negatively to perceived intergroup threats (r = −0.296, *p* < 0.01). In a similar vein, online contact was found to be positively associated with HCNs’ attitudes (r = 0.451, *p* < 0.01), ICC (r = 0.406, *p* < 0.01), and intercultural friendships (r = 0.393, *p* < 0.01) but negatively with perceived threats (r = −0.315, *p* < 0.01). Moreover, ICC and HCNs’ attitudes were significantly correlated with each other (r = 0.583, *p* < 0.01), and both were positively associated with intercultural friendships (r = 0.563, *p* < 0.01) and (r = 0.532, *p* < 0.01), respectively. Perceived intergroup threats, however, were negatively correlated with intercultural friendships (r = −0.368, *p* < 0.01). These findings provided preliminary statistical support for our follow-up model test.

In addition, differences in study variables in terms of demographic variables, including gender (male and female), academic level (undergraduate and graduate), previous overseas education experience (yes and no), and university type (985/211 and non-985/211), were investigated by means of between-subject ANOVAs. Table 2 presents the results. Although there were notable variations in some study variables based on demographic factors, our hypotheses did not primarily focus on these differences. Therefore, we incorporated them as control variables for analysis. 

### 3.3. The Multiple Mediation Effects of ICC, HCNs’ Attitudes, and Perceived Intergroup Threats

**Figure 2:**
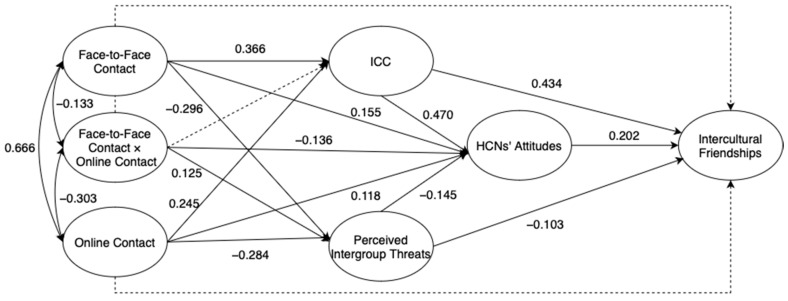
Original model for the direct and indirect effects of Intergroup contact on intercultural friendships (Model A). Dark lines represent statistically significant paths, and dotted lines represent paths that are not statistically significant.

We utilized the technique of structural equation modeling (SEM) to evaluate whether ICC, HCNs’ attitudes, and perceived intergroup threats mediated the relation between intergroup contact and intercultural friendships longitudinally. The interaction term was formed by centering the means of face-to-face and online contact and then multiplying them [66]. In line with the suggested threshold values, the indices of goodness of fit for the multiple mediation model (Model A) based on the association between various variables, as hypothesized, suggested that the data fitted well and was accurately represented (χ^2^ /df = 2.388, *p* < 0.001; CFI = 0.906; IFI = 0.907; TLI = 0.900; SRMR = 0.068; RMSEA = 0.054 (90% CI for RMSEA = [0.052, 0.057])). The model with the completely standardized solution is shown in Figure 2. However, as depicted in Figure 2, the associations between face-to-face/online contact and intercultural friendships and between the interaction terms and ICC/intercultural friendships were not statistically significant in the original model.

**Figure 3:**
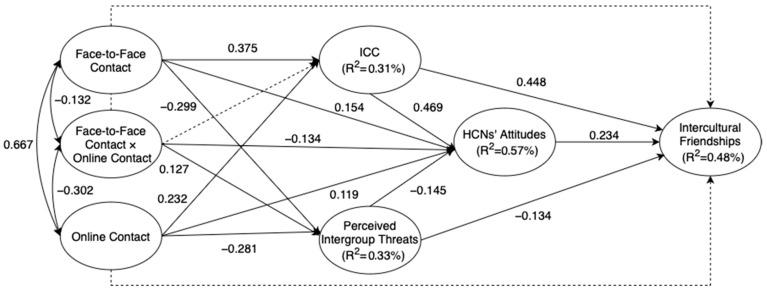
Final model for the direct and indirect effects of intergroup contact on intercultural friendships (Model B).

Statistically speaking, for the purpose of model parsimony, the statistically insignificant pathways were dropped. The subsequent model (Model B) achieved good fit (χ^2^ /df = 2.382, *p* < 0.001, CFI = 0.906, IFI = 0.906, TLI = 0.900, SRMR = 0.068, RMSEA = 0.054 (90% CI for RMSEA = [0.051, 0.057]). The final model presenting the completely standardized solution is displayed in Figure 3. Table 3 presents the results of both unstandardized and standardized parameter estimates as well as related standard errors (S.E.) for the final Model B. In addition, it is also important to note that face-to-face and online contact, taken together, accounted for 31% of the variance in ICC, 33% in perceived intergroup threats, and 57% in HCNs’ attitudes with ICC and perceived intergroup threats combined, respectively. Apart from this, intergroup contact, together with ICC, HCNs’ attitudes, and perceived intergroup threats, accounted for 48% of the variance in intercultural friendships.

As shown in Figure 3, face-to-face contact had a significant positive effect on ICC and HCNs’ attitudes (β = 0.375, *p* < 0.001; β = 0.154, *p* = 0.006) but a negative effect on perceived intergroup threats (β = −0.299, *p* < 0.001). However, the results did not demonstrate a statistically significant association between face-to-face contact and intercultural friendships (β = 0.086, *p* = 0.091). Online contact was found to be positively related to ICC and HCNs’ attitudes (β = 0.232, *p* < 0.001; β = 0.119, *p* = 0.027) but negatively to perceived intergroup contact (β = −0.281, *p* < 0.001) with a large effect size. Likewise, there was also a non-significant correlation between this variable and intercultural friendships (β = −0.032, *p* = 0.0609). We found a significant and positive relationship between ICC and HCNs’ attitudes (β = 0.469, *p* < 0.001) and between ICC and intercultural friendships (β = 0.448, *p* < 0.001) with large effect sizes. Moreover, the variable of HCNs’ attitudes exerted a significant positive influence on intercultural friendships (β = 0.234, *p* < 0.001). Furthermore, a negative association was found between perceived intergroup threats and HCNs’ attitudes (β = −0.145, *p* = 0.002) and between perceived intergroup threats and intercultural friendships (β = −0.134, *p* = 0.004). Therefore, more frequent intergroup contact between HCNs and culturally different others may predict a higher level of ICC, reduced perceived intergroup threats, and more positive intergroup attitudes, which may ultimately and subsequently enhance their willingness to establish friendships with the outgroups. 

Moreover, scholars have recommended assessing mediating effects in SEM through the bootstrapping technique [65]. The utilization of a bias-corrected bootstrap confidence interval to determine the indirect effect can be more informative compared to solely testing the statistical significance of individual pathways in the proposed structural model. They further suggest reporting a 95% CI to test the significance of the mean indirect effect in accordance with the bootstrapping results. If the 95% CI obtained through bootstrapping does not include zero, it proves the statistical significance of the indirect effect [65]. Therefore, bootstrapping analyses with 5000 samples and a 95% CI were included in this study. Table 4 reports the results of the mediation tests.

As presented in the table, the bootstrap 95% CI of the indirect effects of face-to-face contact on intercultural friendships through ICC (β = 0.168, *p* < 0.001, 95% CI = 0.094–0.272), HCNs’ attitudes (β = 0.036, *p* < 0.05, 95% CI = 0.003–0.091), the two mediators combined (β = 0.041, *p* < 0.01, 95% CI = 0.012–0.077), and perceived intergroup threats plus HCNs’ attitudes (β = 0.010, *p* < 0.05, 95% CI = 0.001–0.032) did not cover zero, indicating that these pathways of indirect effects were statistically significant. However, the chain mediation effect of perceived intergroup threats was not statistically significant, as its 95% CI ranging from −0.007 to 0.091 (β = 0.040; *p* = 0.095) included zero. Thus, H2 was partially supported. The same holds true for the mediation effect of perceived intergroup threats in terms of online contact with the 95% CI covering zero (β = 0.038; *p* = 0.095, 95% CI = −0.007 to 0.077). Meanwhile, the indirect effect of online contact on intercultural friendships through HCNs’ attitudes was also insignificant (β = 0.028; *p* = 0.050, 95% CI= 0.000 to 0.071). Nonetheless, all the other indirect effects of online contact on intercultural friendships via ICC (β = 0.104, *p* < 0.01, 95% CI = 0.046–0.172), ICC and HCNs’ attitudes (β = 0.025, *p* < 0.01, 95% CI = 0.006–0.053), and perceived intergroup threats together with HCNs’ attitudes (β = 0.009, *p* < 0.05, 95% CI = 0.001–0.026) were statistically significant. H3 was partially confirmed.

### 3.4. Moderation Analyses

**Figure 4:**
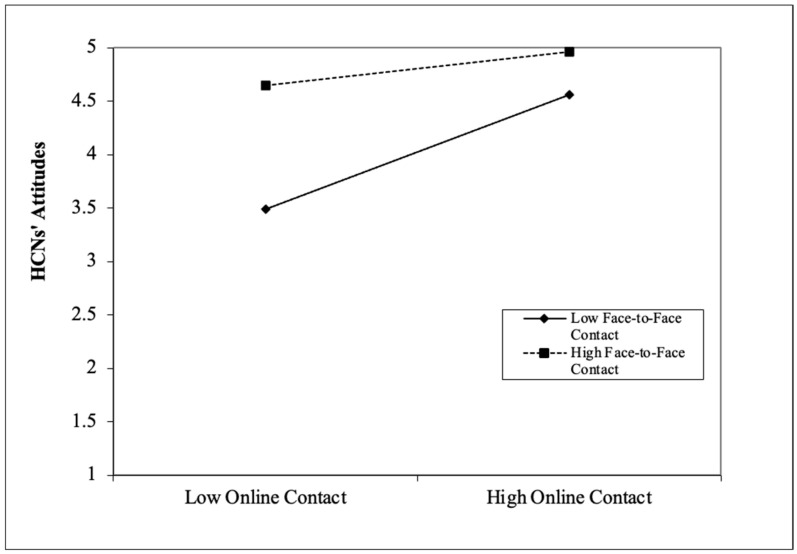
Interaction effects between online and face-to-face contact on HCNs’ attitudes.

**Figure 5:**
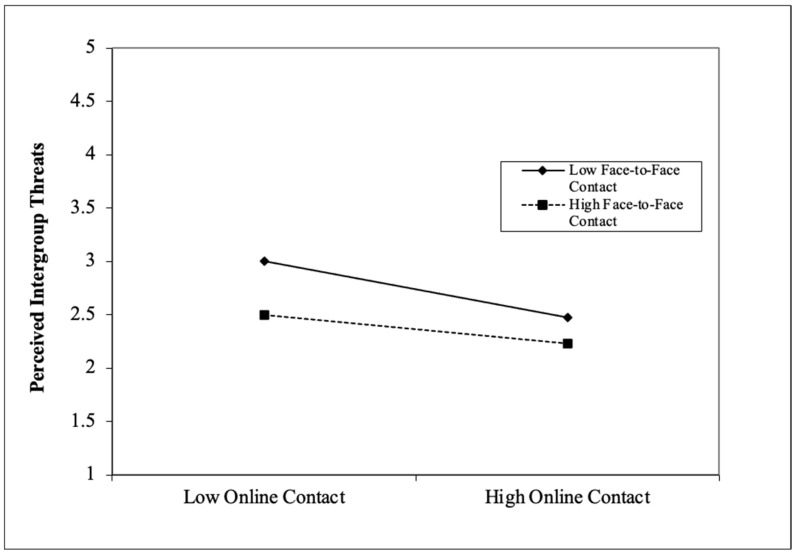
Interaction effects between online and face-to-face contact on perceived intergroup threats.

As presented in Figure 3, the structural model revealed that the interaction between face-to-face and online contact predicted HCNs’ attitudes (β = −0.134, *p* < 0.001) and perceived intergroup threats (β = 0.127, *p* = 0.006). To investigate the interaction effects in more detail, a set of simple slope analyses was conducted to respectively examine the effects of online contact on HCNs’ attitudes and perceived intergroup threats at low (one SD below the mean) and high (one SD above the mean) of face-to-face contact. As depicted in Figure 4 and Figure 5, it was found that online contact was significantly and positively related to HCNs’ attitudes at low levels of face-to-face contact (t = 7.979, *p* < 0.001), whereas the relationship was not significant at high levels of face-to-face contact (t = 1.871, *p* > 0.05) (see Figure 4), thus confirming H1c. In addition, at low levels of face-to-face contact, online contact was more significantly and negatively associated with perceived intergroup threats (t = −5.169, *p* < 0.001) than at high levels of face-to-face contact (t = −2.039, *p* = 0.042) (see Figure 5), supporting H1b. The moderation results indicated that online intergroup contact may function as an effective strategy for improving intergroup attitudes and decreasing perceived intergroup threats, especially when face-to-face contact is scarce or unavailable. 

## 4. Discussion

Forming close friendships with host nationals is essential for international students in their adaptation to college in a foreign context [78], yet little is known about the complex process of relationship formation between HCNs and foreign students, especially in China. Using a sample of Chinese college students as HCNs, the current study combined both mediation and moderation analyses in a single design and constructed a structural model to investigate the roles of intergroup contact, ICC, perceived intergroup threats experienced by Chinese students, and their attitudes toward culturally different students in predicting intercultural friendship formation between the two groups. Several important findings were observed from this model. 

### 4.1. Demographic Variables

In the present study, we found that students from Project 985/211 universities reported significantly more face-to-face and online contact with foreign students. This group was also found to have greater values in ICC, attitudes, and intercultural friendships but lower scores in perceived threats, indicating that they held more favorable attitudes toward the outgroup and were more willing to develop friendships with them. It is important to note that Project 985/211 universities usually host more international students, thus providing increased chances for intergroup interactions and cultural exchanges. Higher levels of intergroup contact would enhance HCNs’ attitudes toward the outgroups and lower possible prejudice and perceived threats. Previous research has found that HCNs interacting with a host of international friends had higher levels of open-mindedness than those without contact, which may play a key role in improving attitudes and increasing willingness for intergroup interactions. Students high in open-mindedness would thus be expected to view international students in a non-prejudicial manner and perhaps be more willing or “open” to interact with them [9].

It is worth noting that graduate students in the study reported significantly higher perceptions of threats than undergraduate students, which resonates with previous research by Spencer-Rodgers and McGovern [12]. Within the realm of academia and highly competitive industries, both domestic graduate students and international students are engaged in a profound struggle to secure admission to esteemed programs, highly sought-after teaching and research assistantships, exclusive fellowships, and lucrative post-graduate employment prospects. In addition, female students, on average, reported having higher levels of perceived threats than males, leading to relatively more negative attitudes toward international students and a decreased tendency to establish intercultural friendships. One plausible explanation for this may be rooted in traditional Chinese cultural norms and gender roles. Females in Chinese cultures may be socialized to be more cautious and less assertive in unfamiliar or potentially threatening situations. This could lead them to perceive greater threats in intercultural interactions. Additionally, familial and educational influences in China may encourage females to prioritize safety and security, potentially leading them to be more vigilant and sensitive to perceived threats [79]. These results contribute to our greater understanding of the roles of gender and educational background in perceived threats and intergroup interactions.

In addition, in the present study, in line with previous research [80], we found that students having previous experience with overseas education performed better than those having no such experience in terms of ICC. This finding indicated that prior intergroup contact experience was closely related to strengthening ICC. It also resonates with previous research by Jackson [81] that focused on the students studying overseas and concluded that oversea experience can be beneficial for improving students’ cultural awareness and intercultural sensitivity as important aspects of ICC. 

### 4.2. Intergroup Contact and Its Relationships with Perceived Intergroup Threats, HCNs’ Attitudes 

Guided by the intergroup contact theory originally proposed by Allport [32], results from the SEM analysis in this study revealed that the two types of intergroup contact between Chinese students and international peers could lead to lower levels of perceived threats and generate more positive attitudes toward the outgroups, similar to findings by Ward and Masgoret [33] and Vezzali et al. [82]. Specifically, Chinese students with more face-to-face and/or online contact with international students may be more likely to have positive intergroup attitudes and reduce perceived intergroup threats, which would then be more willing to form friendships. The findings highlight the essential role of intergroup contact in improving intergroup attitudes and behaviors. 

As we expected, our results also showed that our sample held higher scores on face-to-face contact, indicating that Chinese students were possibly more inclined to have direct contact with international students. The more direct contact HCNs have with international students, the more positive intercultural attitudes they have toward them, the fewer threats they perceive coming from them, and the more willing they become to establish friendly ties and offer them support. This finding is congruent with previous research that found that direct intergroup contact with culturally different others, such as native speakers, can improve students’ intercultural attitudes [83,84] and lower perceptions of intergroup threats and anxiety [37,85]. Meanwhile, the results of this study demonstrated that online contact was also a significant predictor of HCNs’ attitudes and was strongly correlated with decreased perceived intergroup threats. Online contact may act as a crucial avenue for imparting valuable insights to Chinese students, enabling them to grasp the intricacies of international students’ values, beliefs, and convictions infused with cultural codes, thus reducing potential intergroup threats they may feel during their online interactions with international students.

In addition, the moderation analyses yielded impressive results that face-to-face contact acted as a moderator, modifying the relationships between online contact and HCNs’ attitudes and perceived intergroup threats. Specifically, online contact functioned as a positive and negative predictor of HCNs’ attitudes and perceived intergroup threats, respectively. This strong positive relationship with HCNs’ attitudes was only observed at low levels of face-to-face contact and disappeared at high levels of face-to-face contact. Similarly, the negative association with perceived intergroup threats was significantly stronger at low levels rather than at high levels of face-to-face contact. This study was inspired by previous research that has shown that extended contact can improve intergroup attitudes and relationships and reduce perceptions of intergroup threats, especially in cases where face-to-face contact is limited or absent [86]. Later, Vezzali et al. [16], Cao and Meng [87], and Meng et al. [88] substantiated this important finding by extending the outcome variables to attitude, anxiety, prejudice, trust, and empathy. In this regard, our study has contributed new evidence to the existing research by showing that online contact, such as extended contact, can be a helpful strategy when direct face-to-face contact is not sufficient. In more detail, these findings implied that Chinese students low in face-to-face contact in intergroup encounters may instead turn to online contact through a variety of social media platforms, which emerge as the main sources for acquiring cross-cultural knowledge and skills as well as developing close intercultural bonds. Online communication provides individuals with ample time to ponder over their thoughts before exchanging messages, potentially enhancing the quality of communication and promoting mutual relationships due to lowered intergroup anxiety and communication apprehension [89]. 

### 4.3. Intergroup Contact and Its Relationships with ICC

Consistent with prior studies [90,91], our findings showed that both face-to-face and online contact can be effective ways to improve ICC. This finding echoes the argument that students’ ICC can be enhanced through direct and indirect intercultural contact with culturally different others [92]. This is mainly because frequent intergroup contact with students from other countries can effectively promote intercultural communication, increase their knowledge and understanding of different ways of life and perspectives, and foster a deeper level of understanding between individuals of different cultural backgrounds, thus reducing potential perceptions of threats and prejudice. Meanwhile, this process can foster greater empathy, tolerance, and respect for cultural differences as well as provide opportunities for mutual learning and growth [93]. 

Specifically, the results from the present study suggest that participants who engaged in more frequent face-to-face contact with international students reported higher levels of ICC, and face-to-face intergroup contact was a more significant and positive predictor of ICC compared to online contact. In fact, a number of researchers have dedicated their efforts to the study of face-to-face intergroup contact between Chinese and international students, covering topics such as students with intercultural experience, intergroup contact on campus, and studying abroad and exchange programs [80,94]. For instance, the analysis of face-to-face contact on campus indicated that Chinese students in universities show immense interest in intergroup contact with foreign students on college campuses [95]. Furthermore, a combination of interviews and questionnaires has revealed that engaging in study abroad and exchange programs and interacting directly with international students has turned out to strengthen students’ ICC [94].

This study extends the previous findings by Liaw and Johnson [96] and O’Dowd [97] regarding a similarly positive influence on the improvement of students’ ICC through online contact with people from different cultural backgrounds. As revealed by Moon and Park [98] also, frequent use of social media during online contact can significantly aid in acclimatizing individuals to identify and understand the cultural customs, traits, and values of people from different cultural backgrounds. As a result, online contact through social media can greatly enrich one’s cultural awareness, foster empathy and open-mindedness towards different cultures, and cultivate behavioral skills, thus increasing their levels of ICC. In addition, regular exposure to foreign cultural products and items, such as books, music, videos, films, and magazines through social media, is a significant factor in developing one’s ICC [92]. 

### 4.4. ICC, Perceived Intergroup Threats, and HCNs’ Attitudes as Mediators

As the results revealed, ICC functioned as a mediator and had the largest effect sizes compared to the paths mediated by HCNs’ attitudes and perceived intergroup threats. This finding indicates that those students who had more frequent face-to-face and online contact with international students reported higher levels of ICC, which would enhance their tendency to make friends with their foreign peers. This finding is consistent with empirical studies suggesting that both face-to-face and online contact can enhance individuals’ ICC [96], and greater ICC is crucial for friendships to develop [21]. Moreover, ICC was identified to be the strongest predictor, exerting a big effect size on HCNs’ attitudes, supporting Arasaratnam’s ICC model [14]. 

Building on the integrated threat theory [99], this study enriches the literature through the SEM analyses, which also indicate that positive attitudes of HCNs were significantly and positively related to the intentions to establish intercultural friendships with students of different cultures. According to Toh and Srinivas [100], the extent to which HCNs are willing to act as socializers for culturally different others is heavily influenced by the attitudes and actions of both the HCNs and the expatriates themselves as well as how the HCNs perceive the expatriates. This suggests that building positive relationships and fostering mutual understanding between international students and HCNs are key to peaceful and harmonious coexistence. 

As expected, results also provide evidence that increased perceptions of intergroup threats can lead to less positive attitudes toward outgroup members, which would then lower the likelihood of intercultural friendship formation. This finding resonates with a series of the previous literature [33,55]. The data also revealed that some Chinese students perceived foreign students as somewhat of a threat to both their beliefs and values and their social status and economic and educational well-being, which negatively impacted their attitudes and socialization. This finding aligns with several studies that suggest that the perception of foreigners as a threat by host nationals was associated with negative attitudes toward them [101,102]. Perceptions of threats and differences in various aspects (i.e., culture, values) may have an indirect, adverse effect on intergroup attitudes, thus impeding the establishment of friendships and decreasing their willingness to make friends with foreign students. 

However, the results demonstrated that the indirect links of intergroup contact to intercultural friendships via the mediator of perceived intergroup threats were not statistically significant. This relatively weak predictive utility of perceived intergroup threats may be explained by several factors. International students, who are only staying in China for a brief period of time, are not expected to disrupt, undermine, or breach the social and cultural norms of domestic students. On the contrary, it seems that Chinese nationals highly esteem the cultural and intellectual enrichment provided by the international student community. The presence of these students enriches the student body with their diverse racial and ethnic backgrounds while also bringing a wealth of knowledge on different countries, social and political structures, and cultural traditions and practices. Furthermore, as international students are temporary visitors, there is little concern that they would pose a significant economic threat to Chinese students. International students play a significant role in Chinese higher education institutions as they contribute to the maintenance of student enrollments and generate considerable revenue through tuition fees. Additionally, they provide teaching services and add to the intellectual capital of the institutions. 

### 4.5. Intercultural Friendships as the Outcome Variable

As predicted, our results revealed that the outcome variable of intercultural friendships was associated with the two independent variables and three mediators (directly and indirectly) via different pathways. First, ICC was both directly and indirectly related to intercultural friendships as a statistically significant and positive predictor, which means that Chinese students having higher levels of ICC would be more willing to establish friendships with foreign students. This study corroborates the findings of other research [19,46], which proposed that ICC serves as a key factor affecting intercultural friendship formation. Meanwhile, our results showed that greater levels of ICC also predict more positive attitudes, which then function as a mediator to increase students’ tendency to form friendships with international students. This finding expands upon some similar prior studies [27,51], which only focused on some aspects of ICC. 

Second, in line with our expectations, the variable of perceived intergroup threats was found to be negatively associated with intercultural friendships and HCNs’ attitudes toward international students, indicating that Chinese students with more perceptions of threats view and treat foreign students more negatively and would be less likely to make friends with them. This finding is in line with prior research [55,103], which supported a negative association between perceived threats and intergroup attitudes and intercultural interactions and relations. 

Third, consistent with our prediction, we found that the variable of intergroup attitudes of Chinese students was a positive predictor of intercultural friendships, which means that those students with more favorable attitudes toward foreign students showed a greater propensity to build friendships with them. The results suggest that intergroup attitudes are important in affecting HCNs’ intentions to socialize and establish friendships between Chinese and foreign students, confirming the findings of other studies [49,104]. 

Fourth, contrary to our expectations, the direct relationships between face-to-face and online intergroup contact and intercultural friendships were not statistically significant. However, bootstrap analyses of the chain mediation effects suggest that both types of intergroup contact were indirectly and positively correlated with intercultural friendships. The results indicate that intergroup contact alone may be insufficient in increasing Chinese students’ willingness to establish friendships with foreign students, and friendship formation between the two groups involves multiple factors, including higher levels of ICC, reduced perceived intergroup threats, and more positive attitudes. 

Last but not least, the results also indicate that those reported having prior overseas study experience were, to some extent, more likely to initiate a friendship with international students. This finding is in agreement with previous research [18], which indicates that having previous intercultural experience is crucial for establishing and deepening intercultural friendships. 

### 4.6. Limitations, Implications, and Future Research

While this study has contributed to our understanding of how intergroup contact affects intercultural friendships among Chinese and international students, it still has several limitations that can be addressed in future investigations. First, the sample in the study did not include students from Hong Kong, Macao, or Taiwan, and thus, we were unable to present any comparative data for further analysis. These regions are somewhat different from the Chinese mainland in terms of political and economic contexts as well as educational practices [105], which may affect intercultural contact experiences with international students. For future studies, scholars may conduct comparative analyses by involving participants from these regions across various institutional contexts. Second, some of the scales (i.e., ICC, perceived threats) employed in the study were originally designed based on samples outside the context of China and, therefore, may have inadvertently led to measurement errors with the present Chinese student sample. Future studies should consider adapting or developing scales that are culturally and contextually appropriate for the Chinese student population. Third, it is important to note that the findings of the present study are limited to the specific sample investigated in this study, and future studies are recommended to diversify sample sources before making generalizations about the findings. As such, we recommend that interpretations and generalizations of the results in the study be made with caution, and future researchers should carry out similar studies in other countries or regions to control for geographical factors that may have influenced the results. Fourth, the current study was correlational and cross-sectional in nature, which may limit the ability to make causal inferences about the relationships between study variables. Thus, a longitudinal or experimental design is needed to establish the causality of the relationships. Finally, another limitation lies in our failure to examine how host national students interact with their international peers from different countries of origin, diverse racial backgrounds, and different religious affiliations. Future research endeavors may, therefore, delve into the potential influence of these factors on intergroup contact dynamics and the formation of intercultural friendships.

In spite of these limitations, our findings have several implications and strengths. First, the focus of our research is to gain a deeper understanding of the factors that influence the willingness of HCNs to establish friendships with international students in China. By highlighting the crucial role of HCNs, we hope to shed light on the complex dynamics that shape cross-cultural interactions and help facilitate more positive and meaningful relationships between international students and their host communities. Second, this study expands upon the existing theory of intercultural friendships, which has predominantly been examined within Western cultures, by investigating its applicability within the Chinese context. This is a significant contribution as there is a dearth of studies exploring this topic within the Chinese cultural framework. Third, we differentiated the predictive roles of face-to-face and online contact and confirmed their respective functional roles. Fourth, this study adds to the existing literature concerning the contact hypothesis: online contact can be an important predictor for HCNs’ intergroup attitudes and perceived intergroup threats when face-to-face contact is inadequate. 

Moreover, the research results have a series of practical applications. First, on the one hand, given the positive link between face-to-face contact and friendship willingness, institutions should encourage real-world connections. Specifically, at the individual level, instructors should facilitate the inclusion of international students in their classes by establishing group activities that foster cooperation and engagement between domestic and international students. At the institutional level, universities should organize structured events and programs that facilitate socialization, such as cross-cultural workshops, buddy systems, residential programs, and other opportunities for interaction. In addition, more studies should be conducted to evaluate the effectiveness of such programs and to identify specific factors that contribute to their success. On the other hand, institutions should establish more virtual spaces for intergroup interaction. Online forums, discussion boards, or dedicated social media groups can provide avenues for international students and HCNs to engage, share experiences, and communicate across cultures, thereby fostering personal bonds. Second, both domestic and international students are advised to receive targeted intercultural training to expand their culture-specific knowledge and foster intercultural awareness, intercultural sensitivity, and intercultural adroitness given that ICC plays a significant role in affecting intercultural friendship formation. In addition, more research is required as to which aspects of ICC are most relevant in terms of intercultural friendship formation. Third, given the potential deterrent effect of perceived intergroup threats on friendship formation, institutions should initiate awareness campaigns and workshops. These interventions address and reduce such perceptions, creating a more inclusive atmosphere that encourages intercultural interactions. As a final point, it is important to note that there are a series of elements that can affect individuals’ attitudes and their intentions to socialize and develop friendships with international students, such as the salience of group classifications during interactions, national identity, intergroup anxiety, prejudice, stereotypes, and political and multicultural ideology. It is highly recommended that these aspects be systematically examined in future studies.

### 4.7. Conclusions

In conclusion, the findings of this study offer valuable insights with significant implications for both theory and practice in the context of intercultural interactions among Chinese host nationals and international students. From a theoretical perspective, this study enriches the understanding of intergroup contact theory and intercultural friendship formation, particularly within the Chinese cultural context. It highlights the pivotal roles of intergroup contact, ICC, attitudes, and perceived intergroup threats in shaping the willingness of HCNs to form friendships with international students. Additionally, it distinguishes between the effects of face-to-face and online contact, expanding the applicability of contact theory in today’s digitally connected world. In practice, these findings provide actionable recommendations for academic institutions and individuals. Universities should actively promote both face-to-face interactions and virtual spaces for intercultural engagement. Structured programs and events can facilitate real-world connections while online forums and social media groups can foster cross-cultural communication. Moreover, intercultural training for students can enhance ICC and promote intercultural sensitivity, contributing to more positive interactions. In academic management, institutions should consider implementing awareness campaigns and workshops to address perceived intergroup threats and create inclusive environments. These strategies can enhance the overall experience of international students and promote harmonious coexistence on campuses. In summary, this study contributes to the development of strategies and interventions that promote intercultural understanding and positive relationships between HCNs and international students, ultimately enriching the global educational experience.

## Figures and Tables

**Table 1:** Correlations between study variables (*n* = 469).

Variables	M	SD	α	1	2	3	4	5	6
1. HCNs’ Attitudes	4.54	1.16	0.943	1					
2. ICC	4.66	1.03	0.915	0.583 **	1				
3. Intercultural friendships	4.84	1.25	0.921	0.532 **	0.563 **	1			
4. Face-to-Face Contact	4.24	1.55	0.899	0.406 **	0.391 **	0.426 **	1		
5. Online Contact	3.14	1.21	0.938	0.451 **	0.406 **	0.393 **	0.503 **	1	
6. Perceived intergroup threats	3.25	0.85	0.842	−0.352 **	−0.278 **	−0.368 **	−0.296 **	−0.315 **	1

α = Cronbach’s α coefficient. All variables except for online contact (1–5) were measured on a 1–7 scale with a midpoint of 4. ** *p* < 0.01.

**Table 2:** Descriptive statistics by gender, academic level, overseas study experience, and university type and ANOVAs.

	Gender		Academic Level		Overseas Study Experience		University Type	
	Female M (SD)	Male M (SD)	Undergraduate M (SD)	Graduate M (SD)	Yes M (SD)	No M (SD)	985/211 M (SD)	Non-985/211 M (SD)
Face-to-face contact	4.01(1.67)	4.52(1.34)	4.36(1.51)	3.30(1.52)	4.60(1.31)	4.13(1.60)	4.83(1.12)	2.20(1.00)
Statistics	F = 12.81, *p* < 0.001, ηp^2^ = 0.027		F = 23.53, *p* < 0.001, ηp^2^ = 0.048		F = 7.75, *p* = 0.006, ηp^2^ = 0.016		F = 467.58, *p* < 0.001, ηp^2^ = 0.500	
Online contact	2.90 (1.31)	3.46 (0.99)	3.27 (1.15)	2.18 (1.24)	3.42 (0.97)	3.06 (1.26)	3.38 (1.10)	2.33 (1.21)
Statistics	F = 26.64, *p* < 0.001, Hp^2^ = 0.054		F = 42.53, *p* < 0.001, ηp^2^ = 0.083		F = 7.36, *p* = 0.007, ηp^2^ = 0.016		F = 70.58, *p* < 0.001, ηp^2^ = 0.131	
HCNs’ attitudes	4.39 (1.16)	4.72 (1.14)	4.59 (1.16)	4.11 (1.06)	4.72 (1.23)	4.48 (1.13)	4.75 (1.04)	3.78 (1.23)
Statistics	F = 9.64, *p* = 0.002, ηp^2^ = 0.020		F = 8.37, *p* = 0.004, ηp^2^ = 0.018		F = 3.74, *p* = 0.054, ηp^2^ = 0.008		F = 65.39, *p* < 0.001, ηp^2^ = 0.123	
ICC	4.60 (1.02)	4.74 (1.05)	4.68 (1.06)	4.53 (0.820)	4.76 (1.12)	4.63 (1.01)	4.84 (0.91)	4.04 (1.20)
Statistics	F = 1.93, *p* = 0.165, ηp^2^ = 0.004		F = 0.99, *p* = 0.320, ηp^2^ = 0.002		F = 1.26, *p* = 0.263, ηp^2^ = 0.003		F = 54.89, *p* < 0.001, ηp^2^ = 0.105	
Perceived threats	3.39 (0.87)	3.07 (0.81)	3.14 (0.806)	4.10 (0.717)	3.22 (0.94)	3.26 (0.83)	3.13 (0.85)	3.65 (0.74)
Statistics	F = 16.48, *p* < 0.001, ηp^2^ = 0.034		F = 70.61, *p* < 0.001, ηp^2^ = 0.131		F = 0.20, *p* = 0.656, ηp^2^ = 0.000		F = 31.92, *p* < 0.001, ηp^2^ = 0.064	
Intercultural friendships	4.79 (1.28)	4.90 (1.21)	4.82 (1.23)	4.98 (1.42)	4.86 (1.24)	4.83 (1.25)	5.00 (1.08)	4.27 (1.60)
Statistics	F = 0.93, *p* = 0.336, ηp^2^ = 0.002		F = 0.85, *p* = 0.356, ηp^2^ = 0.002		F = 0.05, *p* = 0.819, ηp^2^ = 0.000		F = 29.20, *p* < 0.001, ηp^2^ = 0.059	

**Table 3:** Structural parameter estimates of the SEM model testing the direct and indirect contributions of intergroup contact to intercultural friendships.

Dependent Variables	Predictors	Unstandardized β	Unstandardized S.E.	Standardized β	Standardized S.E.	*p*
Intercultural friendships	HCNs’ attitudes	0.282	0.072	0.234	0.087	0.000
	ICC	0.552	0.073	0.448	0.079	0.000
	Perceived intergroup threats	−0.177	0.062	−0.134	0.077	0.004
HCNs’ attitudes	ICC	0.478	0.053	0.469	0.056	0.000
	Perceived intergroup threats	−0.158	0.051	−0.145	0.068	0.002
	Face-to-face contact	0.105	0.038	0.154	0.062	0.006
	Online contact	0.104	0.047	0.119	0.059	0.027
	Interaction term	−0.080	0.022	−0.134	0.047	0.000
ICC	Face-to-face contact	0.249	0.043	0.375	0.061	0.000
	Online contact	0.200	0.053	0.232	0.059	0.000
Perceived threats	Face-to-face contact	−0.186	0.042	−0.299	0.079	0.000
	Online contact	−0.226	0.055	−0.281	0.077	0.000
	Interaction term	0.070	0.025	0.127	0.074	0.006

S.E. = Standard Error.

**Table 4:** Bootstrap analyses of the statistical significance of indirect effects.

Independent Variable	Mediator Variable	Dependent Variable	Standardized (β)	S.E.	95% CI		*p*
					Lower	Upper	
Face-to-face contact	ICC	Intercultural friendships	0.168	0.045	0.094	0.272	0.000
Face-to-face contact	HCNs’ attitudes	Intercultural friendships	0.036	0.023	0.003	0.091	0.023
Face-to-face contact	perceived intergroup threats	Intercultural friendships	0.040	0.025	−0.007	0.091	0.095
Face-to-face contact	ICC–HCNs’ attitudes	Intercultural friendships	0.041	0.017	0.012	0.077	0.007
Face-to-face contact	perceived intergroup threats–HCNs’ attitudes	Intercultural friendships	0.010	0.008	0.001	0.032	0.024
Online contact	ICC	Intercultural friendships	0.104	0.032	0.046	0.172	0.001
Online contact	HCNs’ attitudes	Intercultural friendships	0.028	0.019	0.000	0.071	0.050
Online contact	Perceived intergroup threat	Intercultural friendships	0.038	0.021	−0.007	0.077	0.095
Online contact	ICC–HCNs’ attitudes	Intercultural friendships	0.025	0.012	0.006	0.053	0.007
Online contact	perceived intergroup threats–HCNs’ attitudes	Intercultural friendships	0.009	0.007	0.001	0.026	0.024

The indirect effect is statistically significant if the bootstrapped 95% confidence interval (CI) does not include 0.

## Data Availability

The data presented in this study are available upon request from the corresponding author. The data are not publicly available due to confidentiality and research ethics.
